# Predictive Value of Cough Frequency in Addition to Aspiration Risk for Increased Risk of Pneumonia in Dysphagic Stroke Survivors: A Clinical Pilot Study

**DOI:** 10.3390/brainsci11070847

**Published:** 2021-06-25

**Authors:** Anna Maria Pekacka-Egli, Radoslaw Kazmierski, Dietmar Lutz, Stefan Tino Kulnik, Katarzyna Pekacka-Falkowska, Adam Maszczyk, Wolfram Windisch, Tobias Boeselt, Marc Spielmanns

**Affiliations:** 1 Department for Pulmonary Medicine and Sleep Medicine, Zürcher RehaZentren, Klinik Wald, 8636 Wald, Switzerland; 2 Department for Neurology and Neurorehabilitation, Zürcher RehaZentren, Klinik Wald, 8636 Wald, Switzerland; dietmar.lutz@zhreha.ch; 3 Department for Neurology and Cerebrovascular Disorders, Poznan University of Medical Sciences, 61701 Poznan, Poland; rkazmierski@ump.edu.pl; 4 Department of Neurology, University of Zielona Gora, 65046 Zielona Gora, Poland; 5 Faculty of Health, Social Care and Education, Kingston University and St George’s University of London, London SW17 0RE, UK; KU70991@kingston.ac.uk; 6 Department for History and Philosophy of Medicine, Poznan University of Medical Sciences, 61701 Poznan, Poland; pekackafalkowska@ump.edu.pl; 7 Department for Methodology, Statistics, and Informatics Systems, Institute of Sport Science, Academy of Physical Education in Katowice, 40065 Katowice, Poland; a.maszczyk@awf.katowice.pl; 8 Department for Pulmonary Medicine, Faculty of Health, University Witten-Herdecke, 58455 Witten, Germany; windischw@kliniken-koeln.de; 9 Department of Pneumology, Cologne Merheim Hospital Kliniken der Stadt Koeln GmbH, 51109 Koeln, Germany; 10 Department of Medicine, Pulmonary and Critical Care Medicine, University Medical Center Giessen and Marburg, Phillips University Marburg, 35037 Marburg, Germany; tobias.boeselt@staff.uni-marburg.de

**Keywords:** cough frequency, dysphagia, aspiration, post-stroke pneumonia, diagnostics, FEES

## Abstract

Background: Post-stroke dysphagia leads to increased risk of aspiration and subsequent higher risk of pneumonia. It is important to not only diagnose post-stroke dysphagia early but also to evaluate the protective mechanism that counteracts aspiration, i.e., primarily cough. The aim of this study was to investigate the predictive value of cough frequency in addition to aspiration risk for pneumonia outcome. Methods: This was a single-center prospective observational study. Patients with first-ever strokes underwent clinical swallowing evaluation, fibreoptic endoscopic evaluation of swallowing (FEES), and overnight cough recording using LEOSound^®^ (Löwenstein Medical GmbH & Co. KG, Bad Ems, Germany ). Penetration–Aspiration Scale (PAS) ratings and cough frequency measurements were correlated with incidence of pneumonia at discharge. Results: 11 women (37%) and 19 men (63%), mean age 70.3 years (SD ± 10.6), with ischemic stroke and dysphagia were enrolled. Correlation analysis showed statistically significant relationships between pneumonia and PAS (r = 0.521; *p* < 0.05), hourly cough frequency (r = 0,441; *p* < 0.05), and categories of cough severity (r = 0.428 *p* < 0.05), respectively. Logistic regression showed significant predictive effects of PAS (b = 0.687; *p* = 0.014) and cough frequency (b = 0.239; *p* = 0.041) for pneumonia outcome. Conclusion: Cough frequency in addition to aspiration risk was an independent predictor of pneumonia in dysphagic stroke survivors.

## 1. Introduction

Stroke is the second leading cause of death worldwide and is associated with many complications, such as long hospital stays and significant health care costs [1]. One common consequence of stroke is dysphagia [2], which leads to an increase in aspiration episodes, subsequent high risk of post-stroke pneumonia, and increased mortality [3,4].

International guidelines recommend overarching aspects of the diagnosis of dysphagia, including early swallow screening, clinical assessment, and—if available—formal instrumental diagnostics, such as a videofluoroscopic swallow study (VFSS) or fibreoptic endoscopic evaluation of swallowing (FEES) [5,6,7]. Non-instrumental assessments are an important base for making a preliminary diagnosis and defining the dysphagia management plan [8]. However, to address aspiration and other physiological problems in the pharyngeal phase of swallowing, a direct observation with instrumental assessment is required [9,10,11]. The implementation of instrumental diagnostics is therefore seen as an important and valuable addition to dysphagia diagnostics [11,12,13]. FEES investigation provides direct information about oropharyngeal secretion management and the effectiveness of clearing mechanisms, such as throat clearing and coughing [14].

Regarding the detection of critical events such as penetrations and aspirations, FEES is at least as efficient as VFSS [15,16]. The occurrence of two or more of the following symptoms may indicate aspiration: dysphonia, dysarthria, lack of gag reflex, inadequate coughing, altered voice quality, and coughing after swallowing [17]. Aspirations can also be asymptomatic, e.g., not accompanied by reflexive protective cough. These so-called silent aspirations increase the risk of pneumonia by a factor of 1.3 compared to aspirations that are accompanied by clinical symptoms [18]. The relationship between coughing disorder, aspiration, and pneumonia has been described in the literature [19]. It is of great relevance to not only diagnose swallowing disorders at an early stage, but to also precisely diagnose the protective mechanism (i.e., cough) that counteracts the risk of aspiration.

In principle, three main aspects of cough are of interest in clinical settings: reflex cough sensitivity, volitional cough strength, and frequency of spontaneous cough [20]. The frequency of coughing is measured primarily in research on respiratory diseases, which cause refractory cough [21] and can inform about the severity of a refractory cough as well as about the etiology of the disease [22]. In connection with dysphagia, most cough research has focused on assessing reflex cough sensitivity or volitional cough strength, but there has been very little research on the role of cough frequency in the context of post-stroke dysphagia [23]. Only very few studies have explored cough frequency in neurological patient groups [24,25]. The incidence of coughs can be measured either by patient self-report, counting of cough episodes by an observer, or by portable cough monitors that are commercially available. One example of such a cough monitor device is the LEOSound system [26]. LEOSound^®^ is a validated, fully automated lung sound monitor, which works as ”long-term stethoscope” and enables continuous objective respiratory sound auscultation for up to 24 h. The device detects, measures, and counts cough events with a sensitivity of 93% and a specificity of 99% [22,26]. The results of long-term cough recordings could potentially provide important information to enhance the results of the dysphagia diagnosis.

The aim of this study was to evaluate the predictive value of cough frequency in addition to aspiration risk for increased risk of post-stroke pneumonia.

## 2. Materials and Methods

This was a prospective observational cohort study. All patients or their legal guardians were informed about the study and gave their written consent. For those patients who were unable to provide their own research consent, the relatives and or legal guardians were approached and they consented on the patients’ behalf.

The local ethics committee approved the study protocol (Kantonale Ethikkommision Zürich, BASEC-No. 2019-01592).

### 2.1. Participants

All patients were referred for neurological rehabilitation from stroke units and acute wards of various regional hospitals to the Zurcher RehaZentren, Klinik Wald, Switzerland, between February and October 2020 following acute onset of stroke.

The inclusion criteria were:

(1) Age between 18–85 years;
(2) First ever stroke (ICD-10-CM Code: Cerebrovascular diseases, I60-I69);
(3) Clinical suspicion of neurogenic swallowing disorder up to 6 weeks post-onset.

Exclusion criteria were:

(1) The presence of neurological condition other than stroke, which could lead to dysphagia;
(2) Known history of swallowing difficulties due to previous stroke;
(3) Any other diseases correlating with increased risk of coughing during night-time measurement (i.e., COPD, asthma);
(4) Pneumonia;
(5) Acute infection;
(6) Requirement for invasive or non-invasive ventilation;
(7) Physical or cognitive impairments leading to limitation in the performance of planed diagnostics (i.e., agitation);
(8) Discharged before assessments could be completed.

### 2.2. Procedures

Within seven days of admission to the Zürcher Reha Zentren, patients underwent complete dysphagia diagnostic procedures. Immediately after enrolment, the patient baseline data was collected from the clinical information system (Phoenix^TM^, CompuGroup Medical AG, Bern, Switzerland).

#### 2.2.1. Functional Independence Measurement (FIM)

The Functional Independence Measure (FIM) was administered to document the severity of physical and psychological disability of our patients at admission and before discharge [27]. FIM is an 18-item tool that quantifies the changes in functional limitations of patients during rehabilitation.

#### 2.2.2. Dysphagia Screening and Clinical Swallowing Evaluation

Risk of dysphagia was first assessed with the Standardized Swallowing Assessment (SSA) using a binary present/absent scoring [28].

Following the results of the SSA, a clinical swallowing evaluation (CSE) combined with the 3 ounce water swallow test [29] was performed according to the in-house protocol based on the screening protocol for neurogenic dysphagia [30].

During both examinations we investigated items relevant for swallowing, such as level of alertness and ability to manage saliva, as well as water swallowing and standardized test meal swallowing (applesauce, banana, and bread).

The aspiration predictors, following Daniels [17], were recorded during the examination using a binary present/absent scoring. The patient was considered at risk of aspiration if two or more of the following aspiration predictors were scored as present: (1) cough after swallow, (2) voice change after swallow, (3) abnormal volitional cough, (4) abnormal gag reflex, (5) dysarthria, (6) dysphonia.

#### 2.2.3. Instrumental Diagnostics of Dysphagia

We performed instrumental diagnostics of dysphagia using FEES (rpSzene^®^, Rehder & Partner Company, Methfesselstrasse, Hamburg, Germany) according to the recommendations by Langmore [31].

An experienced neurologist or a certified speech-language-pathologist carried out the FEES. It was recorded and video-analyzed by the assessor team.

Our in-house protocol includes examination of the condition, function and sensitivity of the anatomical structures included in swallowing, rating of secretion management, and direct swallowing examination. Defined amounts of pudding-like consistency (1 teaspoon = 5 mL), water (1 teaspoon = 5 mL, 1 sip = 10 mL), and solid food (biscuit, 5 g) were offered three times in succession. If any of the consistencies were considered unsafe, we no longer tested the corresponding consistency. All grading procedures during the FEES examination, described below, were carried out according to international standards [32,33,34].

##### Penetration–Aspiration-Scale (PAS)

We graded the severity of penetration or aspiration according to Rosenbek’s Penetration–Aspiration Scale (PAS) [32], with the highest PAS score of all tested consistencies recorded. We classified the results as follows:

(1) PAS I–II: Normal–mild (no aspiration, penetration with clearing);
(2) PAS III–V: Moderate (penetrations);
(3) PAS VI–VIII: Severe (aspirations), with PAS VIII defined as silent aspiration.

##### Rating of Secretion (ROS)

We graded the accumulation of secretion according to the Murray Rating of Secretion Scale (ROS) [33], where the results were classified as follow:

(1) ROS 0: Normal rating;
(2) ROS 1: Any secretions evident upon entry in the protective structures surrounding the laryngeal vestibule that were bilaterally represented or deeply pooled;
(3) ROS 2: Any secretions that changed from a “1” rating to a “3” rating during the observation period;
(4) ROS 3: Any secretions seen in the area defined as the laryngeal vestibule, where pulmonary secretions were included if they were not cleared by swallowing or coughing by the close of the segment.

##### Airway Protection

We graded the airway protection for patterns of tight breath holding (PTBH) following Murray [34] and classified it as follow:

(1) PTBH 1: Breath holding not achieved;
(2) PTBH 2: Transient breath holding with glottis open;
(3) PTBH 3: Sustained breath holding with glottis open;
(4) PTBH 4: Transient true vocal fold closure;
(5) PTBH 5: Sustained true vocal fold closure;
(6) PTBH 6: Transient true and ventricular fold closure;
(7) PTBH 7: Sustained true and ventricular fold closure.

#### 2.2.4. Cough Monitor Assessment

**Figure 1:**
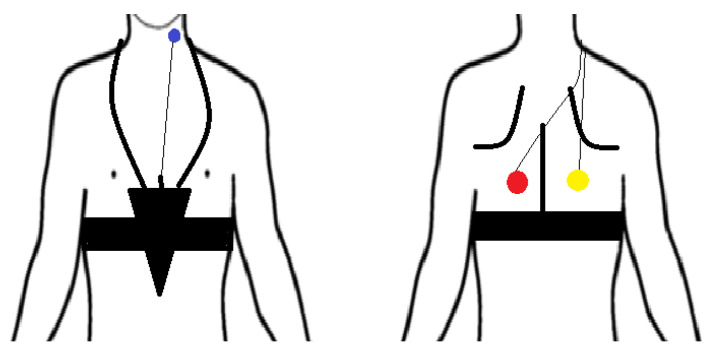
Setup of trachea and lung bio-acoustical microphones for the LEOSound^®^ cough recording.

Following FEES, patients underwent an 8 h nocturnal cough frequency recording with the LEOSound^®^ system. Respiratory sounds were recorded continuously by three small bio-acoustical sensors (microphones) placed on the patient’s back and at the anterior neck of the patient (Figure 1). The placement of the two microphones on the back was chosen after auscultation. Recordings started between 9 p.m. and 10 p.m. After the recordings were completed, recordings were transferred to a computer and analyzed with LEOSound-Analyser^®^ (Löwenstein Medical GmbH & Co. KG, Bad Ems, Germany) software. LEOSound-Analyser^®^ automatically evaluates the data for the presence of respiratory sounds such as wheezing and coughs along with the respiratory rate and stores the results and the raw data in a database. The chief physician (pulmonologist) manually double-checked the accuracy of cough episodes as reported by LEOSound^®^ on the recordings as indicated by LEOSound^®^ and listening to the recording at that time stamp. The chief physician then judged whether the cough sound was actually a cough sound. This was done in approximately 1/5 of patients’ recordings and all sounds matched the characteristics of true cough sounds.

##### Hourly Cough Rate (HCR)

Hourly cough rate (HCR) was presented in a numerical form.

##### Categorized Hourly Cough Events (CHCE)

Categorized hourly cough events (CHCE) were classified as follow:

(1) None: 0 average HCRs;
(2) Minor: 1–2 average HCRs;
(3) Moderate: 3–5 average HCRs;
(4) Distinct: 6 or more average HCRs.

#### 2.2.5. Follow-Up for Incidence of Pneumonia

The occurrence of pneumonia during the neurological inpatient rehabilitation stay was documented from the patient’s medical record on the day of discharge. A diagnosis of pneumonia was determined according to guidelines [35] when all the following occurred:

(1) New lung infiltrates on chest imaging;
(2) Respiratory decline;
(3) Fever;
(4) Productive cough.

### 2.3. Statistical Analysis

All statistical analyses were performed using STATISTICA (Stat Soft, Inc., Tulsa, OK, USA, 2018, version 12). Descriptive statistics were expressed as means ± SD, frequencies, and percentages. The Shapiro–Wilk, Levene, and Mauchly’s tests were used to verify the normality, homogeneity, and sphericity of the sample’s data variances, respectively. Correlation analysis with Pearson’s coefficient was used to determine the strength of the relationship between pneumonia and the variables PAS, ROS, PTBH, HCR, and CHCE. For the variable CHCE, values 1–4 were entered for the respective CHCE categories. Stepwise multiple regression was used to select explanatory variables offering the best predictors for pneumonia in the model construction phase. Ultimately, three predictor variables were used to form a regression model predicting the occurrence of pneumonia. The level of significance for all analyses was set at *p* ≤ 0.05.

## 3. Results

**Figure 2:**
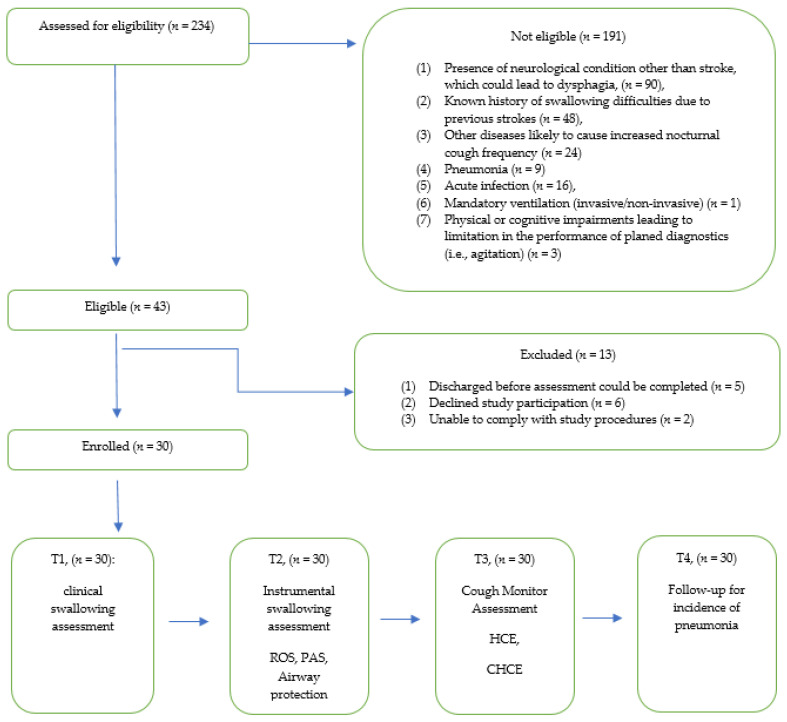
Study flow.

Between February and October 2020, 234 patients with a diagnosis of stroke were admitted for neurological rehabilitation to the Zurcher RehaZentren, Klinik Wald, Switzerland, after hospitalization following acute onset of stroke. Thirty patients met the study inclusion criteria and were enrolled. The flow of participants through the study is shown in Figure 2.

Patients presented to our rehabilitation facility within one month after stroke onset. The study sample consisted of 11 women (37%) and 19 men (63%) with a mean age of 70.3 years (SD ± 10.6; range 47–85). Demographic and clinical patient characteristics, as well as timings of assessments, are summarized in Table 1.

Table 2 presents descriptive statistics of all variables analyzed in this study.

Table 3 shows the results of the correlation analysis for the dependent variable pneumonia.

Correlation analysis showed statistically significant associations between pneumonia and PAS (r = 0.521; *p* < 0.05), HCR (r = 0.441; *p* < 0.05), and CHCE (r = 0.428; *p* < 0.05), with slightly higher correlation coefficients for PAS than for CHCE and HCR.

In the next step, a stepwise regression analysis was performed to determine the most important predictors for pneumonia. Results are shown in Table 4 and Table 5.

The ROS, PTBH, and CHCE variables were outside the model. The analysis showed that ROS, PTBH, and CHCE had no predictive effect for pneumonia.

The regression model was expressed by the following equation:

Y − (Pneumonia = 1) = −4.916 + 0.687 × PAS + 0.239 × HCR

This equation expresses that an increase in PAS by one unit resulted in an increase in pneumonia by 0.687; and a simultaneous increase in HCR by one unit resulted in an increase in pneumonia by 0.239. The stepwise logistic regression analysis showed that the beta value of PAS (0.687) had a greater impact on pneumonia than the HCR variable (0.239).

## 4. Discussion

The aim of this study was to evaluate the predictive value of cough frequency in addition to aspiration risk for outcome pneumonia.

In this prospective cohort of 30 consecutive patients with first-ever acute strokes, we were able to demonstrate statistically significant associations (*p* < 0.05) between pneumonia outcome and PAS ratings according to instrumental swallowing diagnostics using FEES and HCR determined by cough monitor measurement, respectively.

Two possible, somewhat opposing, mechanisms could explain why cough frequency constitutes a predictor for pneumonia in dysphagic stroke patients. On the one hand, abnormally low cough frequency could indicate impaired reflex cough sensitivity and reduced protection of the lower respiratory tract, predisposing patients to higher risk of pneumonia through silent aspiration. On the other hand, abnormally increased cough frequency could indicate frequent protective coughs triggered in response to frequent (micro-)aspiration events. In our data, there was a wide range of hourly cough rates among study participants with poor swallowing function, from normal/unremarkable to abnormally high. For example, in the categorization of hourly cough rates (none, minor, moderate, and distinct) among the 15 participants with PAS ratings of 7–8, eight had moderate or distinct hourly cough events, while seven had minor events or none. Our analysis supports the second of the above-mentioned possible mechanisms, i.e., increased cough frequency as an indicator and ”warning sign” of aspiration and increased risk of pneumonia.

The literature offers very limited data on the role of cough frequency in the context of impaired swallowing and pneumonia risk in stroke and other neurological patient groups. Hadjikoutis et al., 2000, presented data on self-reported occurrence of coughing and choking episodes in patients with motor neuron disease [24]. In their population, coughing and choking episodes were common but infrequently associated with overt chest infection. Kulnik et al., 2015, in their longitudinal one-month observational study, noted that average nocturnal cough frequency as measured with the Leicester Cough Monitor [36] in acute-stroke survivors was higher at baseline and reduced over time, with wide individual variability [25]. Their results support our suggestion that frequent protective coughs triggered in response to frequent aspiration events may constitute the underlying mechanism by which higher cough frequency indicates increased pneumonia risk. This also aligns with the general tendency of spontaneous recovery of dysphagia following acute stroke; if frequent coughing occurs as a response to (micro-)aspiration events, abnormally high cough frequency would resolve as dysphagia improves over time.

Three of our patients with abnormally high hourly cough rates (39, 15, and 6 each) were using ACE inhibitors, which are known to induce cough in a proportion of patients [37]. For medical reasons, ACE inhibitors could not be discontinued to monitor cough in this study. The cough-inducing effect of ACE inhibitors affects on average only 10% of patients and generally wanes several days or weeks after the medication has been introduced [37]. Nevertheless, future research should account for the potential influence of ACE inhibitor use on cough frequency.

To the best of our knowledge, this is the first study to report an association between hourly cough frequency and the incidence of pneumonia in dysphagic patients after stroke. We were able to show, that in our study cohort, cough frequency measured with LEOSound^®^ cough monitors had a similar predictive value for the diagnosis of pneumonia as PAS according to FEES.

The current coronavirus pandemic has imposed numerous restrictions on clinical procedures that carry a high risk of virus transmission, such as dysphagia and cough diagnostics. Instrumental swallowing examinations have only been used for the evaluation of the severely affected [38], and dysphagia has been evaluated mainly through clinical assessment. Observer-based assessment of cough has been restricted due to the risk of exposure to aerosols for both staff and patients [39]. In this current situation, and potentially in health care systems in which access to instrumental diagnostic equipment is only possible to a limited extent, systems like LEOSound^®^ could potentially offer a safe alternative to instrumental diagnostic for the estimation of pneumonia risk, while reducing the potential for exposure to aerosols and secretions.

There are practical aspects to our research: continuous monitoring of cough frequency during the night, when the risk of silent aspiration is often higher, may be an effective and, in contrast to FEES, non-invasive method. If the results obtained in this study can be validated in future clinical studies, this may lead to enhanced preventive procedures in stroke and rehabilitation facilities.

### Strengths and Limitations

The strengths of this study were the prospective study design with consecutive recruitment and the long-standing experience of the clinical team in conducting clinical and instrumental swallowing diagnostics. The analysis was limited due to the small sample size and the study should be viewed as exploratory.

Despite this limitation, it may be concluded that in our study cohort HCR values recorded by LEOSound^®^ had similar predictive value for pneumonia outcome as PAS according to FEES. Based on these findings, it is warranted to conduct further studies to investigate the following research question: is higher nocturnal cough frequency in patients with dysphagia after stroke an indicator of the cough defense mechanisms against aspiration and therefore a warning sign for pneumonia? In order to conduct a definitive study, our data could be used to conduct a prospective sample-size calculation, and eligibility criteria would have to be carefully considered. In future studies, it would be helpful to include repeated assessments of cough frequency to examine possible relationships to change in the swallowing function and to determine the stability of HCR measurements over time. Additionally, all three relevant aspects of cough—frequency, reflex cough sensitivity, and peak cough flow—should be investigated as potential predictors of pneumonia, especially in dysphagic patients who are highly exposed to pneumonia.

## 5. Conclusions

Increased cough frequency measured with a nocturnal cough monitor was an independent predictor of pneumonia in dysphagic stroke patients. Further studies are required to confirm the clinical utility of cough monitoring in this patient group. As a non-invasive assessment method with low infection risk, cough monitoring may offer a viable alternative or complementary method to instrumental diagnostics of swallowing.

## Figures and Tables

**Table 1:** Participant characteristics.

Characteristic	Sample (*n* = 30)
Sex (*n*, %)	
Female	11 (36.7)
Male	19 (63.3)
Age, years (mean, SD)	70.3 (10.6)
Height, cm (mean, SD)	170.4 (10.6)
Weight, kg (mean, SD)	70.8 (13.2)
BMI (mean, SD)	24.3 (3.7)
Stroke etiology (*n*, %)	
Ischemic	26 (86.7)
Hemorrhagic	4 (13.3)
Stroke lesion site (*n*, %)	
Left	14 (46.7)
Right	13 (43.3)
Bilateral	1 (3.3)
Medulla	2 (6.7)
FIM (mean, SD)	
Total score	64.7 (23.7)
Cognitive sub-score	22.8 (10.9)
Motor sub-score	41.9 (17.6)
Aphasia (*n*, %)	12 (40.0)
ACE inhibitors (*n*, %)	3 (10.0)
Nasogastric tube (*n*, %)	5 (16.7)
PEG/PEJ tube (*n*, %)	7 (23.3)
Length of stay, days (median, range)	38 (5, 86)
Pneumonia incidence during rehabilitation stay (*n*, %)	10 (33.3)
Admission to SSA, days (median, range)	0 (0, 1)
SSA to CSE, days (median, range)	0 (0, 3)
CSE to FEES, days (median, range)	2 (0, 5)
FEES to overnight cough monitor (LEOSound),	
days (median, range)	0 (0, 2)

BMI, body mass index; FIM, Functional Independence Measure; ACE, angiotensin-converting enzyme; PEG, percutaneous endoscopic gastrostomy; PEJ, percutaneous endoscopic jejunostomy; SSA, Standardized Swallowing Assessment; CSE, clinical swallowing evaluation; FEES, fibreoptic endoscopic evaluation of swallowing.

**Table 2:** Results for descriptive statistics for PAS, ROS, PTBH, HCR, CHCE, and pneumonia.

Variables	Sample (*n* = 30)
PAS (*n*)—(mean, SD)	6 (2.300)
ROS (*n*)—(mean, SD)	0 (0.681)
PTBH (*n*)—(mean, SD)	5 (1.545)
HCR (*n*)—(mean, SD)	4 (7.718)
CHCE (des)—(mean, SD)	2 (2.355)
Pneumonia (bi)—(mean, SD)	0 (0.479)

PAS, Penetration–Aspiration Scale; ROS, rating of secretion; PTBH, patterns of tight breath holding; HCR, hourly cough rate; CHCE, categorized hourly cough event; bi, binary; des, descriptive; *n*, numeral.

**Table 3:** Results of the correlation analysis between PAS, ROS, PTBH, HCR, CHCE, and pneumonia.

Variables	Pneumonia (r-Value)
PAS	**0.521**
ROS	0.352
PTBH	−0.186
HCR	**0.441**
CHCE	**0.428**

PAS, Penetration Aspiration Scale; ROS, rating of secretion; PTBH, patterns of tight breath holding; HCR, hourly cough rate; CHCE, categorized hourly cough events; statistically significant value in bold.

**Table 4:** Results of stepwise regression analysis for dependent variable pneumonia—variables in the model.

Variables in Model	Beta	OR	*p* *	−95% CL	95% CL
Constant	−4.916	0.007	**0.011**	0.000	0.332
PAS	0.687	1.987	**0.014**	1.146	3.445
HCR	0.239	1.270	**0.041**	1.011	1.595

PAS, Penetration Aspiration Scale; HCR, hourly cough rate; * statistically significant value in bold.

**Table 5:** Results of stepwise regression analysis for dependent variable pneumonia—variables outside the model.

Variables Outside Model	Beta	OR	*p*	−95% CL	95% CL
ROS	0.052	0.487	0.303	0.118	2.843
PTBH	0.082	0.157	0.411	0.776	2.631
CHCE	0.105	0.740	0.102	0.457	1.133

ROS, rating of secretion; PTBH, patterns of tight breath holding; CHCE, categorized hourly cough event.

## Data Availability

Data supporting the reported results can be accessed by corresponding with the authors.

## Clinical Trial Registration Information

Deutsches Register Klinischer Studien; Registration number: DRKS00025011.
